# Supplementary material for: Malleability and Physicochemical Properties of Industrial Sheet Margarine with Shea Olein after Interesterification
Source: Foods. 2022 Nov 11;11(22):3592. doi: 10.3390/foods11223592 (PMC9689388; doi:10.3390/foods11223592)
Supplement: Supplementary file 1 [file foods-11-03592-s001.zip › foods-1968303-supplementary.pdf]

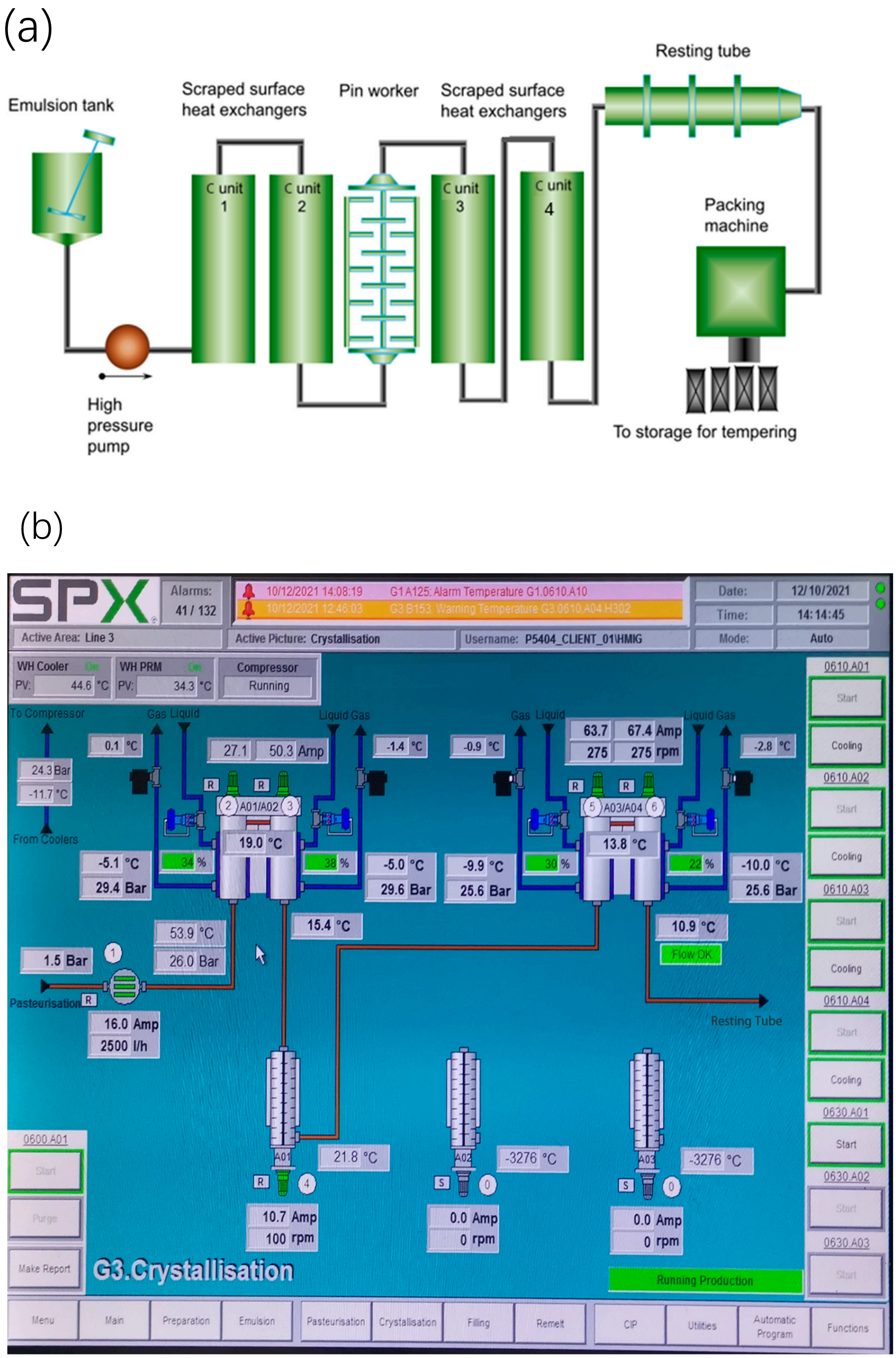

Fig. S1 Processing Parameters of Industrial Sheet Margarine (ISM)

(a) is the schematic of the margarine plant SPX Nexus 244 LC system.

(b) is the detailed processing parameters of production.
